# Earlier-Season Vegetation Has Greater Temperature Sensitivity of Spring Phenology in Northern Hemisphere

**DOI:** 10.1371/journal.pone.0088178

**Published:** 2014-02-05

**Authors:** Miaogen Shen, Yanhong Tang, Jin Chen, Xi Yang, Cong Wang, Xiaoyong Cui, Yongping Yang, Lijian Han, Le Li, Jianhui Du, Gengxin Zhang, Nan Cong

**Affiliations:** 1 Institute of Tibetan Plateau Research, Chinese Academy of Sciences, 4A Datun Road, Chaoyang District, Beijing, China; 2 Center for Environmental Biology and Ecosystem Studies, National Institute for Environmental Studies, Onogawa, Tsukuba, Japan; 3 State Key Laboratory of Earth Surface Processes and Resource Ecology, Beijing Normal University, Beijing, China; 4 Department of Geological Sciences, Brown University, Providence, Rhode Island, United States of America; 5 College of Life Sciences, University of Chinese Academy of Sciences, Beijing, China; 6 State Key Laboratory of Urban and Regional Ecology, Research Center for Eco-Environmental Sciences, Chinese Academy of Sciences, Beijing, China; 7 State Key Laboratory of Vegetation and Environmental Change, Institute of Botany, Chinese Academy of Sciences, Beijing, China; 8 School of Geographical Science and Planning, Sun Yat-Sen University, Guangzhou, China; 9 Department of Ecology, College of Urban and Environmental Sciences, Peking University, Beijing, China; Tennessee State University, United States of America

## Abstract

In recent decades, satellite-derived start of vegetation growing season (SOS) has advanced in many northern temperate and boreal regions. Both the magnitude of temperature increase and the sensitivity of the greenness phenology to temperature–the phenological change per unit temperature–can contribute the advancement. To determine the temperature-sensitivity, we examined the satellite-derived SOS and the potentially effective pre-season temperature (*T*_eff_) from 1982 to 2008 for vegetated land between 30°N and 80°N. Earlier season vegetation types, i.e., the vegetation types with earlier SOS_mean_ (mean SOS for 1982–2008), showed greater advancement of SOS during 1982–2008. The advancing rate of SOS against year was also greater in the vegetation with earlier SOS_mean_ even the *T*_eff_ increase was the same. These results suggest that the spring phenology of vegetation may have high temperature sensitivity in a warmer area. Therefore it is important to consider temperature-sensitivity in assessing broad-scale phenological responses to climatic warming. Further studies are needed to explore the mechanisms and ecological consequences of the temperature-sensitivity of start of growing season in a warming climate.

## Introduction

Spring phenology is one of the vegetation traits that is most responsive to climate [1]. Changes in start of vegetation growing season (SOS) that occur at a broad spatial scale can alter vegetation activity and ecosystem functions during the entire year that follows [2]–[4]. Further, they can affect land–atmosphere energy and carbon budgets [5], [6] and even the regional climate [7], [8]. Therefore, it is essential to understand the spring phenological response of vegetation to climate in order to evaluate and model ecosystem dynamics in climate change studies [9]–[11].

SOS can be studied at either vegetation or species scales. Vegetation-scale studies of SOS, which use greenness vegetation indices derived from remote sensing data at coarse (hundreds to thousands of square meters) spatial resolution, can provide spatially continuous information over large areas [12]. Species-scale studies, in contrast, rely on direct human observations of the timing of discrete events such as leaf-out or flowering of individual plants [13]. Studies at both scales have reported pronounced changes in the SOS in northern middle and high latitudes in response to accelerated warming since the early 1980s [14]–[20]. SOS at a vegetation scale has been related to spatial and temporal changes in spring temperature [21]–[23], and it also is affected by other environmental factors such as precipitation, winter temperature, and photoperiod [16], [24], [25]. Even though temperature is considered the major determinant of greenness phenology, little is known about the temperature-sensitivity of SOS at vegetation scale, which is the phenological change per unit temperature [26]. Such broad-scale information on temperature-sensitivity is urgently needed, however, for predicting the effects of climate warming on vegetation dynamics.

Studies based on direct human observations have reported different phenological responses to spring temperature [18], [27] caused by differences in sampled species [28], water availability [29], photoperiod [30], and winter temperature [31]–[33]. Moreover, data sets compiled at both continental and global scales suggest that the species-level phenological response to temperature is stronger in those species that leaf out or flower earlier [20], [26], [34], [35]. On the basis of these species-level findings, we hypothesized that at a vegetation scale, an earlier SOS would be associated with higher temperature-sensitivity. To test this hypothesis, we first investigated whether the vegetation with earlier growing season (earlier mean SOS over 1982–2008) had a greater SOS advancement from 1982 to 2008 than the vegetation with later growing season. Then we examined whether the temperature-sensitivity of vegetation that usually starts growing season earlier was higher than that of vegetation that usually starts growing season later.

## Materials and Methods

### Ethics Statement

This study is based on data derived from satellite remote sensing technique and climate model. The data are freely available to the public.

We first used the normalized-difference vegetation index (NDVI), a vegetation greenness index, to determine annual SOS during 1982–2008 for all vegetated lands in the Northern Hemisphere temperate and boreal regions (30°N–80°N) [17]. We then determined the duration of the pre-season period during which temperature was significantly related to SOS [17], based on the 1.875°×1.91° daily air temperature at 2-m in the NCEP/DOE reanalysis II data set [36], [37], and defined the mean temperature during this period as the potentially effective pre-season temperature. Finally, we examined temporal trends in the potentially effective pre-season temperature (defined in section 2.2) and SOS during 1982–2008 in relation to the mean SOS (SOS_mean_). Here the trend of SOS is the slope in the linear regression of SOS against year, and so does the trend of the potentially effective pre-season temperature. SOS_mean_ is the mean SOS over the period 1982–2008, and is used to indicate the time when vegetation usually starts growing season. The temperature-sensitivity of SOS was calculated for each pixel as phenological change per unit temperature using linear regression. The Student’s T-test was used the test the significance of the temporal trends and coefficients in linear regressions in the analyses.

### 2.1. Determination of the Start of the Growing Season from Satellite Imagery

The NDVI data set that we used was prepared by the Global Inventory Monitoring and Modeling Study and was produced at a spatial resolution of 8 km by the 15-day maximum-value composition technique (i.e., by selecting the highest NDVI value from each period of 15–16 days) by using observations made by the Advanced Very High Resolution Radiometer (AVHRR) instrument on board the NOAA satellite series. This NDVI data set has been corrected for instrument calibration, viewing geometry, volcanic aerosols, and other effects not related to vegetation changes [38]–[40].

Winter and early-spring NDVI values in the study area are often negatively biased by the effect of snow cover. To reduce snow contamination, we replaced any winter (1 January to 1 March) NDVI value that had been marked as affected by snow in the flag file for data quality with the mean of uncontaminated winter values (December–1 March) for that pixel from the closest 5 years (e.g., for 1986, the years from 1984 to 1988). This step was implemented separately for the periods from 1982 to 2000, when the data were from AVHRR2 sensor, and from 2001 to 2008 (AVHRR3 sensor) in case different sensitivities of the sensors to bright backgrounds led to different instrumental errors. We further excluded those pixels with four consecutive NDVI values flagged as snow-contaminated during the period from the fifth to the seventeenth 15-day period (March to 15 September). Then, to further reduce contamination by clouds, snow, and ice, we applied the Savitzky–Golay filtering procedure to each annual NDVI cycle [41]. After that, to focus on the areas with vegetation and seasonality, a pixel is included in further analysis if it meets the following 3 requirements. First, the average of NDVI from June to September should be higher than 0.10. Second, the annual maximum NDVI should occur within July-September. Third, the average value of NDVI for July-September should be higher than 1.2 folds of the average NDVI of November-March. Finally, we defined SOS as the first day of the year (DOY) that the NDVI increased by 20% of its annual range [42]: that is, NDVI_ratio_ >0.2, where



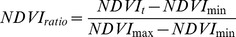
(1)


NDVI*_t_* is the NDVI value at a given time *t*, and NDVI_max_ and NDVI_min_ are respectively the maximum and minimum NDVI values in the annual NDVI cycle. The threshold (20%) was determined by *Yu et al.* [42] from *in situ* observations. It is notable that there are many methods to define SOS from annual NDVI [43], and the interannual changes in SOS derived from these are similar among each other [43], [44]. We thus chose this threshold method because of its low computation cost.

### 2.2. Pre-season Temperature

In the temperate Northern Hemisphere, the vegetation SOS is primarily determined by the spring temperature in the months period preceding the event (henceforth referred as pre-season), and higher pre-season temperatures may advance the SOS [17], [37], [45], [46]. These suggest that there should be negative inter-annual correlation between the pre-season temperature and SOS. Moreover, the duration of the pre-season period during which temperature primarily influences the SOS varies spatially, ranging from a few weeks to four months in the Northern Hemisphere [16], [17], [47]. In this study, we therefore determined the duration of this period for each pixel by performing a correlation analysis between the SOS and temperature (Figure S1). The temperature data were re-sampled to the spatial resolution of NDVI before analysis. First, for each pixel we calculated the mean temperature for each of 36 periods with durations ranging from 15 to 120 days (i.e., 15, 18, 21, …, 120, here the 3-day step is used to smooth potential extreme temperature) preceding the SOS_mean_ during 1982–2008. Then, using linearly detrended values [17], we calculated Pearson’s correlation coefficient between the 27-year time series of SOS and the mean temperature during each of these 36 periods, thus obtaining an array of 36 correlation coefficients for each pixel. We defined the duration of the pre-season period for which the mean temperature has the minimum coefficient (closest to –1.0) among the 36 periods. Then, the potentially effective pre-season temperature (*T*_eff_) in the pixel was determined as the mean temperature during the pre-season period of the selected duration for that pixel for each year between 1982 and 2008.

## Results

### 3.1 Trends in Effective Pre-season Temperature and SOS

**Figure 1:**
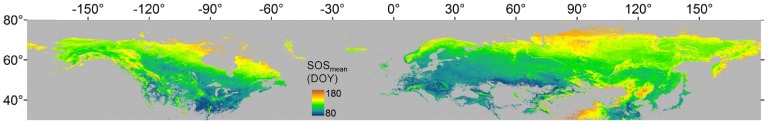
Spatial distribution between 30°N and 80°N of the start of the growing season SOS (SOS_mean_) as day of year (DOY), averaged over 1982–2008.

We first characterized the spatial distribution pattern of SOS_mean_ during 1982–2008 (Figure 1). At a hemispherical scale, the SOS_mean_ was generally later at higher latitudes and altitudes. In the middle latitudes, SOS_mean_ tended to be earlier in southeastern North America, southeastern China and Japan, western and southern Europe. In North America, the SOS_mean_ became later toward the northwest from late March to early June, except in the northeast, where the land surface usually turned green in late June. In Eurasia, the SOS_mean_ occurred in late March in western Europe and in mid-June in northeastern and northern Russia. In East Asia, the SOS_mean_ became clearly later northwestward from southeastern China to central Eurasia, and northward from Japan and the Korean Peninsula to Siberia.

**Figure 2:**
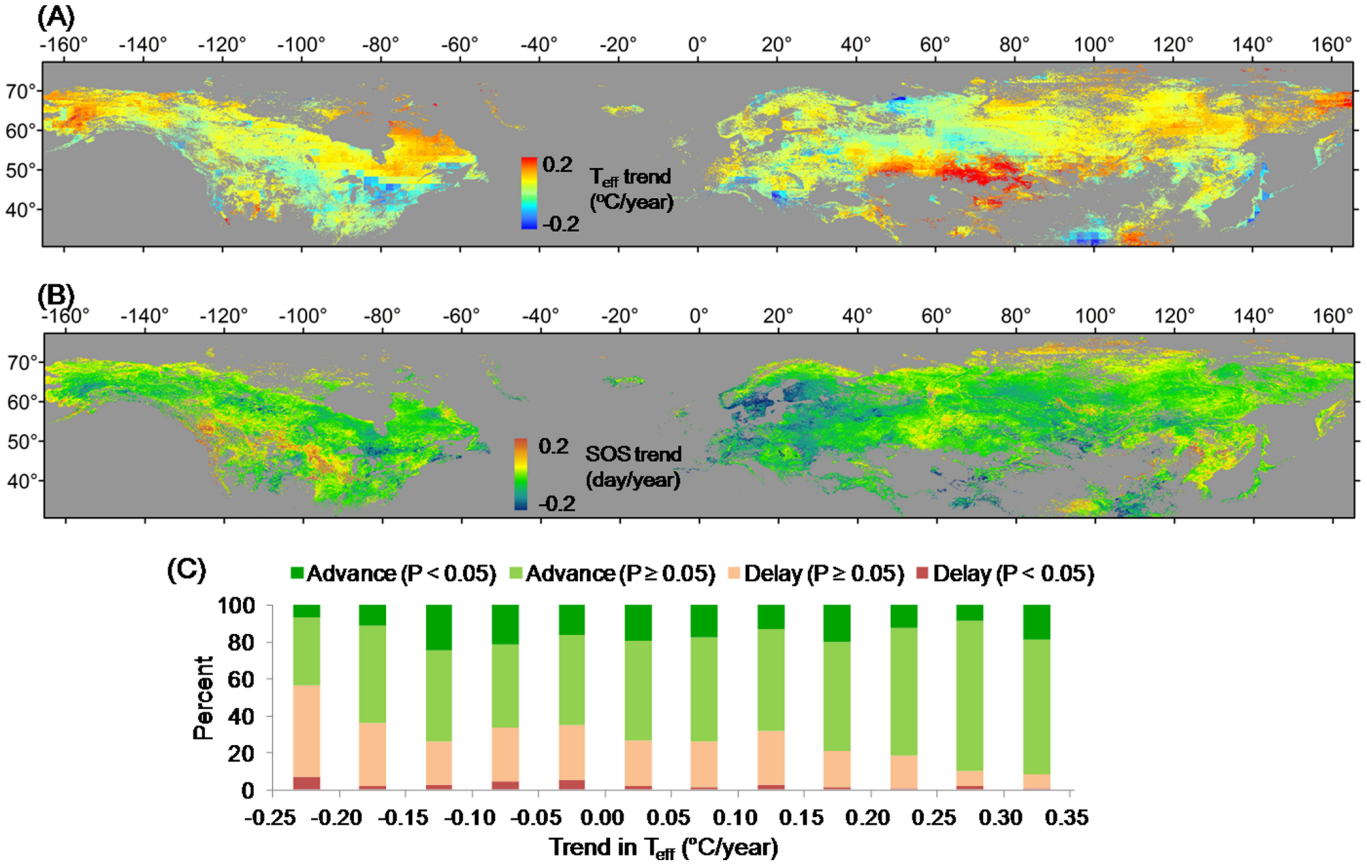
Spatial distribution of the rate of change of the potentially effective pre-season temperature (*T*_eff_) from 1982 to 2008 (A). Spatial distribution of the rate of change of the SOS from 1982 to 2008 (B). Percentages of negative and positive SOS change rates in relation to the rate of change in *T*_eff_. “Advance” means negative SOS change rates (the SOS tends to become earlier), and “Delay” means a positive SOS change rates (the SOS tends to become later) (C). Only pixels with a negative SOS–*T*_eff_ correlation that is significant at *P*<0.10 level are included. Here *T*_eff_ is the mean temperature of the pre-season period that has most negative correlation coefficient (closest to −1) with SOS (see details in section 2.2).

*T*_eff_ increased from 1982 to 2008 in 79.5% of the pixels with a significantly negative SOS–*T*_eff_ correlation and 30.0% of the *T*_eff_ increase was significant at *P*<0.10 level. *T*_eff_ increased at a rate faster than 0.1°C/year in three regions: central and southern Russia south of the Kara Sea; the circumpolar Arctic region (consisting of parts of Greenland, northern Canada, and Alaska and the northeastern edge of Russia); and the area around the Black Sea and the Caspian Sea (Figure 2A). Moreover, *T*_eff_ tended to increase in eastern Canada, eastern China, and in part of central Eurasia.

Among the pixels in which *T*_eff_ increased and the SOS–*T*_eff_ correlation was significantly negative at *P*<0.10 level, SOS advanced in 71.8% of the pixels during 1982 to 2008 (Figure 2C). SOS advanced mainly in a belt northwestward from the Great Lakes region to south central Alaska in North America, in most of Europe, in west central Russia, and in central and eastern Asia (Figure 2B).

**Figure 3:**
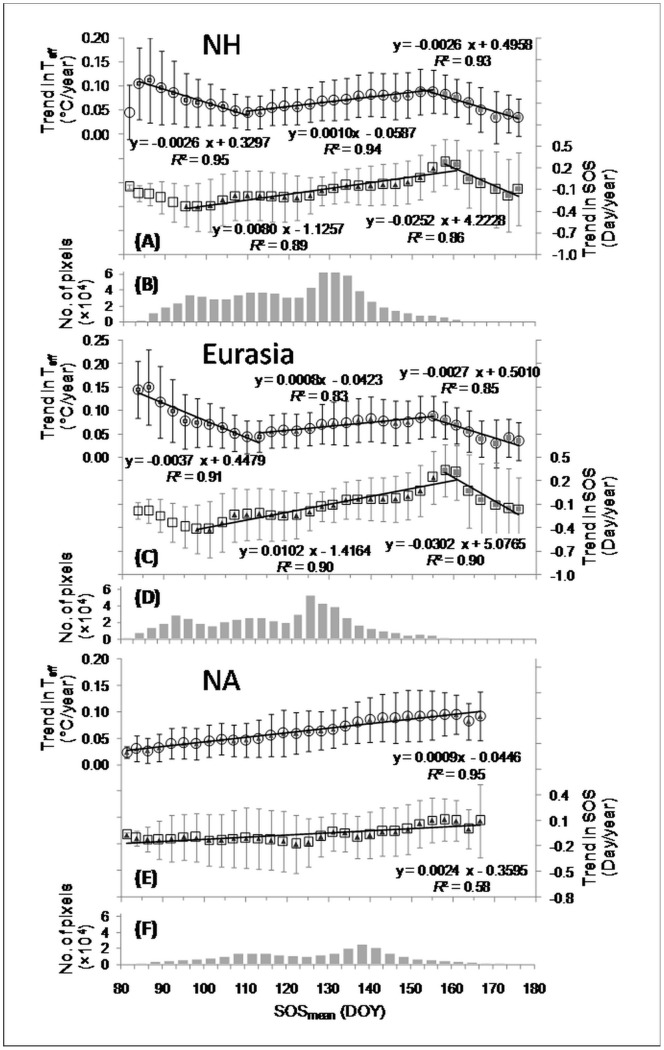
Rates of change in the potentially effective pre-season temperature (*T*_eff_) and in the SOS in relation to the SOS_mean_ during 1982–2008, in the Northern Hemisphere (NH), Eurasia, and North America (NA), respectively. All regressions shown are significant (*P*<0.01) ((A) (C) and (E)). The number of pixels in each SOS_mean_ bin, in the NH, Eurasia, and NA, respectively ((B), (D), and (F)). Only pixels with a positive change rate in *T*_eff_ and a negative SOS-*T*_eff_ correlation that is significant at *P*<0.10 level are included. Here *T*_eff_ is the mean temperature of the pre-season period that has most negative correlation coefficient (closest to −1) with SOS (see details in section 2.2).

In those areas with increasing *T*_eff_ and a negative SOS–*T*_eff_ correlation that is significant at *P*<0.10 level (about 62.5% of all vegetated pixels), the rate first decreased significantly (*P*<0.01) from 0.10°C/year in areas where the SOS_mean_ was DOY 86 to about 0.045°C/year where the SOS_mean_ was DOY 110; it then increased steadily (*P*<0.01) to nearly 0.09°C/year in areas where the SOS_mean_ was DOY 155, and finally decreased to about 0.025°C/year where the SOS_mean_ was DOY 175 (Figure 3A). That is, where SOS_mean_<DOY 110 or SOS_mean_>DOY 155, the pre-season warming was more intensive (i.e., *T*_eff_ was higher) in those areas with earlier SOS_mean_, but where DOY 110< SOS_mean_<DOY 155, the pre-season warming was more intensive in those areas with later SOS_mean_. When DOY>155, it has less than 1000 pixels in NH, as well as in both Eurasia and North America (Figures 3B, 3D, and 3F).

A greater *T*_eff_ increase did not necessarily result in greater SOS advancement (Figure 3A). Where DOY 82< SOS_mean_<DOY 95, SOS tended to advance as the SOS_mean_ became later, with an advancing rate of 0.33 day/year at SOS_mean_ = 95, and a delaying rate at 0.10 day/year at SOS_mean_ = 82. Where DOY 95< SOS_mean_<DOY 160, in contrast, SOS tended to delay as the SOS_mean_ became later, with an advancing rate of 0.33 day/year at SOS_mean_ = 95 and a delaying rate of 0.20 day/year at SOS_mean_ = 160. Where SOS_mean_>DOY 160, SOS tended to advance as the SOS_mean_ became later, with an advancing rate of about 0.20 day/year at SOS_mean_ = 175. Consequently, in areas where DOY 92< SOS_mean_<DOY 160, SOS advanced more strongly (6.5 days/month = 0.008 days/year×30 days/month×27 years) as SOS_mean_ became earlier during 1982–2008.

The changes in the SOS and *T*_eff_ trends in relation to the SOS_mean_ in the Eurasia (Figure 3C) were similar to those for the Northern Hemisphere (Figure 3A),butthe *T*_eff_ trends continuously increased with SOS_mean_ in the North America (Figure 3E). The SOS trends also continuously increased with SOS_mean_, from being negative (i.e., advance of SOS from 1982 to 2008) in the areas with SOS_mean_<DOY140 to being positive in the areas with larger SOS_mean_. Nevertheless, the patterns still revealed that a greater *T*_eff_ increase did not necessarily result in greater SOS advancement.

### 3.2. Correlation between SOS and Pre-season Temperature

**Figure 4:**
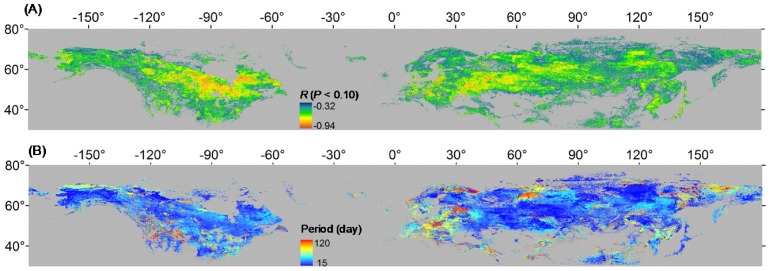
Spatial distribution of the correlation coefficients between the detrended SOS and the detrended pre-season temperature (*T*_eff_) (A). Spatial distribution of the duration of the pre-season period used to calculate *T*_eff_ (B). Only pixels with a correlation that is significant at *P*<0.10 level between the SOS and *T*_eff_ are colored. Here *T*_eff_ is the mean temperature of the pre-season period that has most negative correlation coefficient (closest to −1) with SOS (see details in section 2.2).

**Figure 5:**
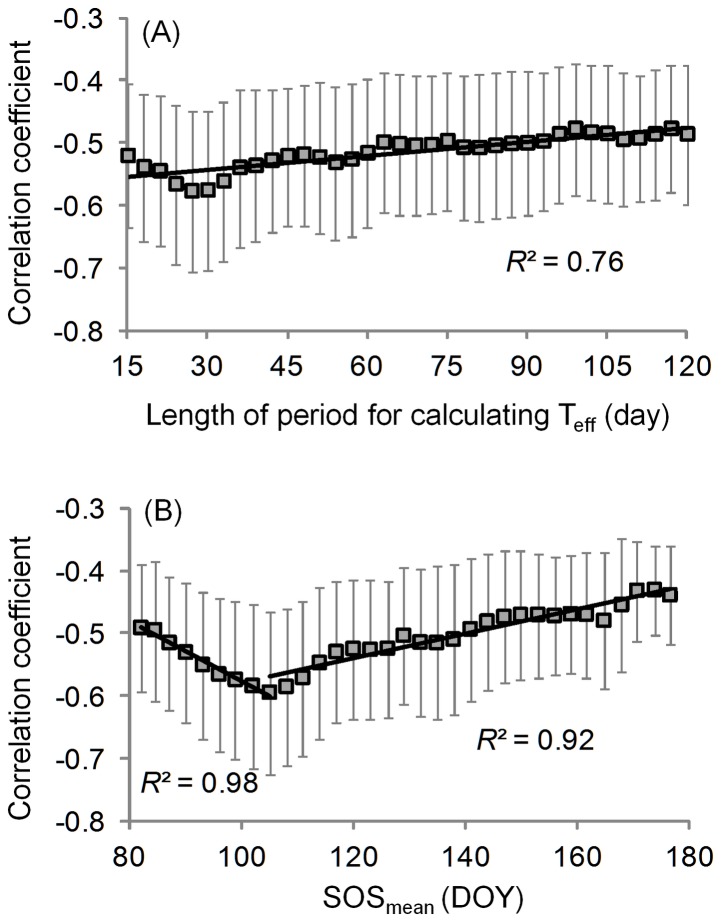
Correlation coefficients between the detrended SOS and detrended effective pre-season temperature (*T*_eff_) in relation to (A) the duration of the period used for calculating *T*_eff_ and (B) SOS_mean_. The regressions are significant (*P*<0.01) level. Only correlation coefficients that are significant at *P*<0.10 level were included. Here *T*_eff_ is the mean temperature of the pre-season period that has most negative correlation coefficient (closest to −1) with SOS (see details in section 2.2).

About 78.6% of all pixels exhibited a negative correlation with significance at *P*<0.10 level between the detrended SOS and the detrended *T*_eff_, with Pearson’s correlation coefficient, *R*, ranging from –0.94 to –0.32 (Figure 4A). Correlations between *T*_eff_ and SOS were stronger in the Great Lakes region and central North America than in other parts of North America. In Eurasia, stronger correlations were found in central Europe and in western and central Russia. In most areas, the length of pre-season period with temperature that has most negative correlation coefficient with SOS was shorter than 2 months (Figure 4B). Furthermore, we found stronger correlations (i.e., more negative *R* values) between *T*_eff_ and SOS in areas where the duration of the pre-season period used to calculate *T*_eff_ was shorter (Figure 5A). The most negative values of *R* (average, –0.57) were associated with a period of about 30 days.

Under the assumption that at a landscape scale, plants did not modify their spring phenological strategy during 1982–2008, we used SOS_mean_ to represent the SOS resulting from long-term local adaptation at a given location. The negative correlations between detrended *T*_eff_ and detrended SOS became stronger as SOS_mean_ increased from DOY 82 (*r* = –0.52) to DOY 105 (–0.61), and then became weaker toward SOS_mean_ = DOY 175 (–0.47) (Figure 5B).

### 3.3 Spatial Pattern of Temperature-sensitivity of the Phenological Response

**Figure 6:**
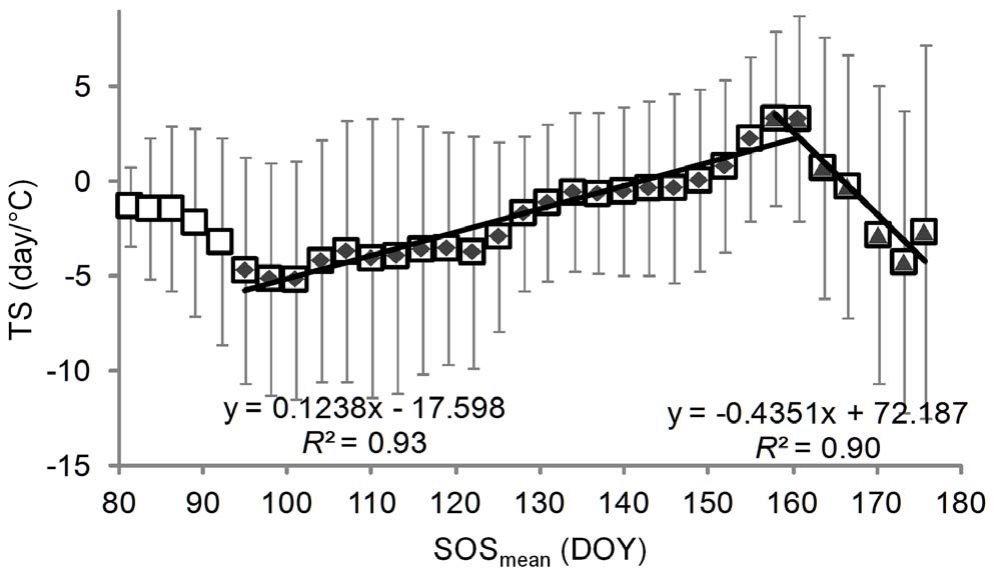
Sensitivity of SOS to *T*_eff_ in relation to SOS_mean_ during 1982–2008. Both regressions shown are significant (*P*<0.01). Only pixels with positive change in *T*_eff_ and a negative SOS–*T*_eff_ correlation that is significant at *P*<0.10 level are included. Here *T*_eff_ is the mean temperature of the pre-season period that has most negative correlation coefficient (closest to −1) with SOS (see details in section 2.2).

**Figure 7:**
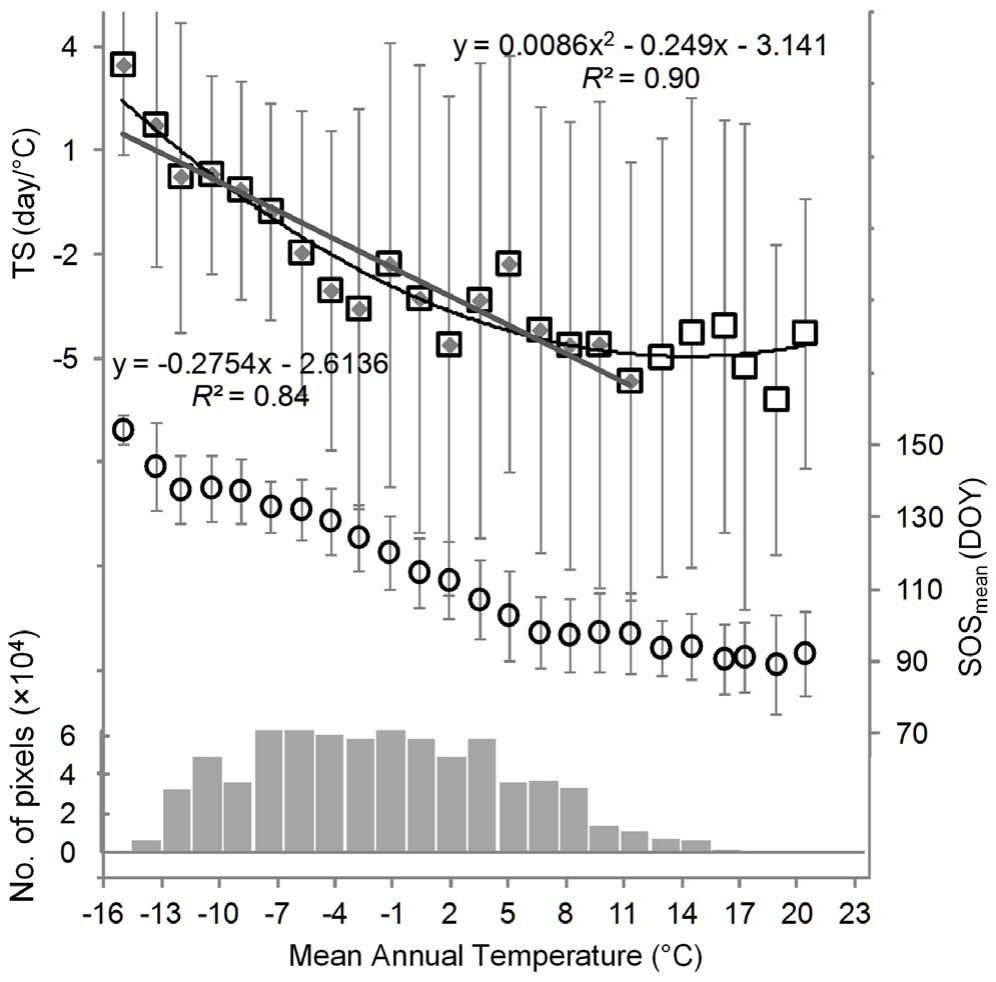
Temperature-sensitivity of the SOS (upper), the SOS_mean_ during 1982–2008 (middle), and the number of pixels (bottom) in relation to mean annual temperature during 1979–2008. We used the 30-year mean annual temperature to represent the climatic temperature condition. Both regressions shown are significant (*P*<0.01). Only pixels with a positive change in *T*_eff_ and a negative SOS–*T*_eff_ correlation that is significant at *P*<0.10 level are included. Here *T*_eff_ is the mean temperature of the pre-season period that has most negative correlation coefficient (closest to −1) with SOS (see details in section 2.2).

*T*_eff_ increased at the fastest rate in areas where SOS_mean_ is in late March, late May, or early June. However, the greatest advancement in SOS was found in areas where SOS_mean_ is in early April (Figure 3A). One cause of this discrepancy might be the sensitivity of the phenological response to increases in *T*_eff_ (Figure 6). The temperature-sensitivity, defined as the ratio of the change in SOS to the change in *T*_eff_, was most negative (–6.0 day/°C, here more negative value indicates greater advancement of the SOS for each degree of increase in *T*_eff_) in areas with SOS_mean_ around early April, and as SOS_mean_ became later, from DOY 95 to DOY 160, the temperature-sensitivity increased. Since warmer areas usually have earlier SOS_mean_, we also examined the pattern of the temperature-sensitivity in relation to mean annual temperature. The temperature-sensitivity shows more negative values in areas wither higher mean annual temperature, in areas where mean annual temperature lower to about 10°C (Figure 7).

**Figure 8:**
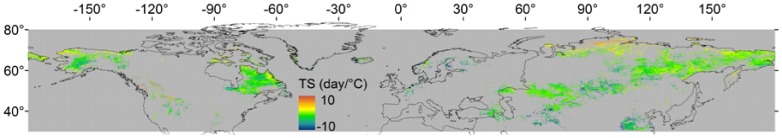
Temperature-sensitivity (TS) of the SOS in pixels between 30°N and 80°N with an increase in pre-season temperature (*T*_eff_) that is significant at *P*<0.10 level and an SOS–*T*_eff_ correlation that is significant at *P*<0.10 level. Here *T*_eff_ is the mean temperature of the pre-season period that has most negative correlation coefficient (closest to −1) with SOS (see details in section 2.2).

We next examined the geographical distribution pattern of temperature-sensitivity in areas with warming trend that is significant at *P<*0.10 level (Figure 8). In those areas, the most negative temperature sensitivities were mainly in central Eurasia, southern Russia, and in a few pixels south of Hudson Bay and in western and northern Europe.

**Figure 9:**
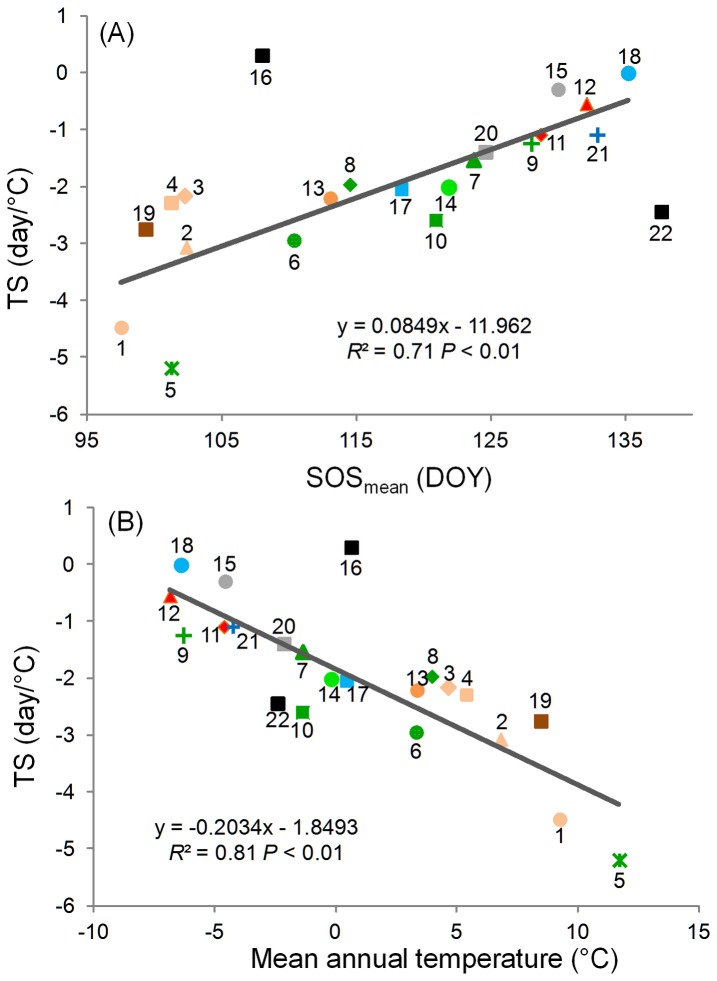
Temperature-sensitivity of the SOS for different land-cover classes in relation to (A) the mean SOS (SOS_mean_) and (B) mean annual temperature. See Table S1 for descriptions of land-cover classes C1–C23. C22 and C16 were not included in the regressions. The classes are C1, irrigated croplands; C2, rainfed croplands; C3, cropland dominated mosaics; C4, natural vegetation dominated mosaics; C5, closed to open broadleaved evergreen or semi-deciduous forest; C6, closed broadleaved deciduous forest; C7, open broadleaved deciduous forest; C8, closed needleleaved evergreen forest; C9, open needleleaved deciduous or evergreen forest; C10, closed to open mixed forest; C11, mosaic forest or shrubland/grassland; C12, mosaic grassland/forest or shrubland; C13, shrublands; C14, grasslands; C15, sparse vegetation; C17, forest/woody wetland; C18, grassland/woody wetland; C19, urban areas; C20, bare areas; C21, water bodies; C22, permanent snow and ice; C16, flooded broadleaved forest; C23, no classification data (not shown in figure). The two classes excluded from the regression are flooded broadleaved forest (C16, which occupies only 11 out of a total of 227,387 pixels) and snow and ice (C22, 2080 pixels). The values are the average of those in pixels with a negative SOS–*T*_eff_ correlation that is significant at *P*<0.10 level and a *T*_eff_ increase that is significant at *P*<0.10 level. Here *T*_eff_ is the mean temperature of the pre-season period that has most negative correlation coefficient (closest to −1) with SOS (see details in section 2.2).

We further found higher temperature-sensitivities associated with land-cover classes with earlier SOS_mean_. Temperature-sensitivity became more negative at a rate of 2.55 ( = 0.0849×30) days/°C per month as the SOS_mean_ of the land-cover classes became earlier (Figure 9A and Table S1). The greatest temperature-sensitivity (most negative values, from –4.5 to –2.2 day/°C) was exhibited by croplands and urban areas, with irrigated croplands (C1, Figure 9A) showing the most sensitive response to changes in *T*_eff_, followed in order of decreasing response by rainfed croplands (C2), artificial and urban areas (C19), and natural vegetation–cropland mosaics (C3 and C4). Among forest land covers, temperature-sensitivity ranged from –4.9 to –1.2 day/°C, and broadleaved forests (C5 and C6) were more sensitive than needleleaved forests (C8 and C9), and mixed forest (C10) showed an intermediate response. The temperature-sensitivity of shrublands (C13) was –2.2 day/°C, which was close to that of grasslands (C14), –2.0 day/°C. Sparse vegetation (C15) and grassland/woody wetland (C18) seemed insensitive to changes in *T*_eff_. Because the land cover of some pixels would have been misclassified [48], we also examined temperature-sensitivity changes in relation to SOS_mean_ by using two other land-cover products, based on images obtained by NOAA-AVHRR and by the Système Pour l’Observation de la Terre (SPOT-VGT). These data also showed that biomes with earlier SOS_mean_ were more temperature sensitive (*R* ^2^ = 0.76 and *R* ^2^ = 0.75, both *P*<0.01, Figure S2 and Tables S2 and S3).

The more temperature sensitive vegetation types (land-cover classes) were usually distributed in warmer areas: a 1°C increase in mean annual temperature corresponded to a sensitivity increase of about 0.20 day/°C (Figure 9B). To determine whether this trend reflected the magnitude of warming during the study period, we examined the relationship between temperature-sensitivity and the rate of change in *T*_eff_ (Figure S3), but the regression result was not significant (*P*>0.42).

## Discussion

### 4.1. Temperature-sensitivity of SOS at Broad Scale

Remote sensing techniques have been widely used to assess broad-scale changes in the onset of spring greenness in response to temperature. Most of these studies emphasized the role of pre-season temperature increases in advancing the onset of spring greenness at a broad scale [10], [21], [45], [49]–[51], but they revealed little about the temperature-sensitivity of the onset of spring greenness at a landscape or biome scale. In this study, we showed that temperature-sensitivity also plays an important role in shaping the response of the spring greenness onset to warming temperatures (Figures 3 and 6). This result suggests that greater magnitude of advance in spring greenness onset does not simply indicate large temperature increase. Furthermore, at a broad spatial scale, the temperature-sensitivity of the onset of spring greenness also depends on the dates: the temperature-sensitivity is higher in warmer areas where the SOS_mean_ is earlier. This information may provide reference for evaluations of the phenology module in ecosystem models.

### 4.2. The Possible Role of Spring and Winter Temperature in the Pattern of Temperature-sensitivity

Why phenological temperature-sensitivity differs among species or locations is still a matter of debate [43], [52]–[54]. Because few broad-scale data are available, to explain the temperature-sensitivity pattern of plant spring phenology (i.e., the association of higher sensitivity with an earlier mean onset time or with warmer areas) at vegetation and biome scales, we examined the results of species-level studies [20], [26], [34], [35]. They also showed higher temperature-sensitivity associated with earlier mean onset of spring phenological events such as leafing and budburst. We thus try to explain our findings with help of studies at species scale (also because little candidate mechanisms at vegetation scale are available ).

Species-scale studies have suggested that the SOS reflects the growth response to forcing temperatures (spring temperatures that force growth after dormancy has been released, similar to *T*_eff_ in this study) and to chilling temperatures (winter low temperatures necessary to release dormancy) [55], who defined the state of forcing (*S*_f_) as the sum of daily forcing rates,


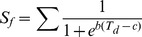
(2)

where *T*_d_ is daily mean temperature and *b* and *c* are empirically determined parameters (*b* <0, *c* >0). SOS occurs when the critical state of forcing (F*) is reached (i.e., when *S*_f_ = F*). Therefore, if F* is constant in a given area among different years, then in a year with higher *T*_d_ during the forcing period the SOS tends to be earlier, because *S*_f_ increases with *T*_d_, as indicated by the positive value of 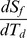:



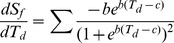
(3)Furthermore, a higher value of 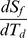 suggests a higher sensitivity of the SOS response to the forcing temperature. With regard to spatial variation, 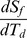 varies with *T*_d_, and it increases with *T*_d_ when *T*_d_<*c* (see Figure S4 for an example). Thus, an area with higher *T*_d_ during the forcing period may show higher temperature-sensitivity if *b* and *c* are fixed and *T*_d_<*c*. Because pre-season temperatures tend to be higher in warmer areas [56], Eqs. (2) and (3) may explain why forest vegetation types with an earlier SOS_mean_ (or in warmer areas) show higher temperature-sensitivity (Figure 8). However, it remains to be confirmed whether this model proposed by *Chuine* [55] can exactly explain the patterns of temperature-sensitivity at a broad spatial scale or those of other vegetation types.

The SOS of vegetation may also be regulated by winter temperatures. The greater temperature increases in winter and early spring tended to be larger at higher latitudes [57], thus have shortened the chilling period and possibly causing it to be insufficient to meet the chilling requirement of some vegetation types. As a result, the forcing temperature requirement for the onset of greenness would increase [31], [55], [58]–[60], thus may delay the SOS date even if the spring temperature was increased [25], [33], [61], [62]. Therefore, vegetation in colder areas that experienced larger increases in winter temperatures may show less sensitivity to pre-season temperature.

### 4.3 Other Influencing Factors of SOS

In addition to temperature which is the primary factor, other environmental factors may also influence the SOS of vegetation and contribute to the lower temperature-sensitivity in areas with a later SOS_mean_. First, plants with a later growing season might use more static cues such as the photoperiod and thus be less sensitive to temperature [30], [53], [63], [64]. Second, in arid and semiarid areas, low water availability due to insufficient precipitation can delay the SOS [16], [65] and even lower the temperature-sensitivity of plants [43]. In this study, the higher temperature-sensitivity found in warmer areas with an earlier SOS_mean_ may be due in part to higher precipitation in those areas (Figure S5). Third, in colder areas, the higher frost risk may also prevent plants from closely tracking the temperature cue [66], [67] and probably result in lower temperature-sensitivity. In addition, differences in effects of CO_2_ fertilization and nitrogen depositions may also have contributed to the spatial variations of SOS response to temperature [68].

The pattern of temperature-sensitivity may also be related to vegetation type. Agricultural vegetation has long been artificially adapted to the thermal environment, partly through management of the timing of sowing and transplanting, and thus may be more sensitive to temperature variation. The onset of greenness in grasslands and shrublands, where conditions are dryer, might also be more affected by precipitation [65]. In contrast, forests are generally distributed in relatively wet areas, where the role of precipitation may be relatively less important than that of temperature in the timing of the greenness onset. Furthermore, because higher inter-annual variability of *T*_eff_ in colder areas (Figure S6) is associated with lower temperature-sensitivity of forests (Figure 9), it is possible that forests that are adapted to unstable temperature conditions [53], [54] may be less sensitive to changes in temperature. In addition, the temperature-sensitivity was averaged from the entire study area and may not precisely reflect the spatial pattern in specific locations. For example, in Central Europe, the grassland flushes earlier than forest. This suggests that the temperate grasslands may have larger temperature-sensitivity than forest in Central Europe.

Greenness phenology at a broad spatial scale is influenced by multiple factors, including the timing of the fulfillment of the winter chilling requirement, warm temperatures in the spring, water availability, photoperiod, solar radiation, and human activities [16], [25], [42], [65], [69], [70]. Yet it is not clear how these multiple environmental factors drive phenology, especially at a landscape scale [71], [72]. Moreover, little is known how the effects of these factors change across observation scales and across taxa [71]. The pattern of temperature-sensitivity revealed in this study should be further examined by observing phenology–environment relationships at various spatial scales [10], [72]–[74].

### 4.4 Some Practical Notes

A recent study [43], using the same NDVI dataset, showed similar patterns in the relationships between SOS and pre-season temperature and the temperature-sensitivity, despite the differences in the threshold used to extract the SOS. The results of Cong et al. (2013) thus indicate that using a different threshold such as 50% will not change our conclusion about the temperature-sensitivity in this study. This should be attributed to the fact that the interannual variations in SOS retrieval are mostly determined by the shifts of NDVI profile [4] and thus necessitates the attention of carefully coping with the noises in the NDVI data [75], [76].

We further used the monthly air temperature data at spatial resolution of 0.5°×0.5° prepared by the Climate Research Unit (CRU) [77] to perform the analysis, in case there is uncertainty in the air temperature from the reanalysis dataset. As show in Figure S7A, the pattern of trend in SOS for 1982–2008 in relation to SOS_mean_ based on the CRU temperature is similar to that based on reanalysis temperature (Figure 3A), but there is slight difference in the magnitude of *T*_eff_ trend for the areas with SOS_mean_ between DOY 95 and DOY 110 (Figures 3A and S7A). Consequently, there is also generally similar pattern for temperature-sensitivity in relation to SOS_mean_ between the two temperature datasets, except the slight difference in the areas with SOS_mean_ between DOY 95 and DOY 110 (Figure 3B and Figure S7B). At biome scale, the vegetation types with earlier SOS_mean_ also exhibited significantly higher temperature sensitivity, except the lower coefficient of determination (Figure 9 and Figure S8). These differences might be caused by the uncertainty in the reanalysis data, the interpolation procedure of the CRU data, or the difference in spatial and temperature resolutions between the two temperature datasets, which should be addressed in future research.

The Global Inventory Monitoring and Modeling Study NDVI dataset in the above analysis has been comprehensively evaluated and applied for different studies before [17]. The new and updated version of it, named NDVI3 g, is supposed to be very much improved and was produced for period from 1982 to 2010 [78], [79]. To test how robust our conclusions are when changing to a different vegetation dataset, we re-performed the analyses with the NDVI3 g (1982–2010) and CRU temperature data, and found similar results as those based on the earlier NDVI dataset (1982–2008) (Figures S7–S8 Vs. Figures S9–S10). Nevertheless, the causes of the slight difference need further identification when the technique details are published (manuscript in plan, Pinzon et al. *Revisiting error, precision and uncertainty in NDVI AVHRR data: development of a consistent NDVI3* *g time series*, as indicated in http://www.mdpi.com/journal/remotesensing/special_issues/monitoring_global).

## Conclusions

We showed a spatial pattern in which vegetation in areas with an earlier SOSmean showed greater advancement of the SOS during 1982–2008. Furthermore, the temperature-sensitivity of the SOS was higher in areas with an earlier SOSmean. Our results indicate that, in addition to the magnitude of temperature increase, the sensitivity of the SOS response to temperature should also be considered in assessments of broad-scale greenness phenological shifts under climatic warming. Future studies should examine the consequences and mechanisms of the different temperature sensitivities of SOS.

## Supporting Information

Figure S1 **A schematic diagram indicating the determination of the duration of the preceding period with the potentially effective pre-season temperature.** The x-axis gives the duration of the period preceding SOS_mean_ of which the inter-annual variations in temperature are correlated (y-axis gives the correlation coefficient) to the inter-annual variations in SOS. In this case, the mean temperature of the 66-day period (the blue vertical line) preceding SOS_mean_ is determined as the potentially effective pre-season temperature.
(TIF)

Figure S2 **Temperature-sensitivity (TS) of the start of growing season (SOS) for different land-cover classes in relation to the mean SOS during 1982–2008 (SOS_mean_).** Land covers are based on images obtained by NOAA-AVHRR and by SPOT-VGT (see Tables S2 and S3 for details). Land-cover types 1 and 21 in (B) were not included in the regression calculation. The values are the average of those in pixels with a significantly (*P*<0.10) negative SOS–T_eff_ correlation and a significant (*P*<0.10) T_eff_ increase.
(TIF)

Figure S3 **Relationship between the temperature-sensitivity (TS) of the SOS and the rate of change of the pre-season temperature (** ***T*** **_eff_).** See Figure 8 and Table S1 for the land-cover types. The values are the average of those in pixels with a significantly (*P*<0.10) negative SOS–*T*_eff_ correlation and a significant (*P*<0.10) *T*_eff_ increase.
(TIF)

Figure S4
**Relationship between** 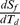 **and** ***T*** **_d_. In this example,** ***b*** **= –0.2 and** ***c*** **= 30.**
(TIF)

Figure S5 **Mean annual, March–May (MAM), and April–June (AMJ) precipitation during 1982–2008 in relation to (A) SOS_mean_ and (B) mean annual temperature.** Only pixels with a positive change in *T*_eff_ and a significantly (*P*<0.10) negative SOS–*T*_eff_ correlation are included. Monthly temperature and precipitation data are from the CRU TS 3.2 data set (Mitchell TD and Jones PD, 2005, An improved method of constructing a database of monthly climate observations and associated high-resolution grids. *Int J Climatol* 25∶693–712.).
(TIF)

Figure S6 **Relationship between the standard deviation (S.D.) of** ***T*** **_eff_ and mean annual temperature for the forest land-cover classes.** See Figure 9 for the land-cover types. The values are the average of those in pixels with a significantly (*P*<0.10) negative SOS–*T*_eff_ correlation and a significant (*P*<0.10) *T*_eff_ increase.
(TIF)

Figure S7 **Similar as** **Figure 3A** **, but using temperature extracted from the CRU (Climate Research Unit) dataset (A).** Similar as Figure 6, but using temperature extracted from the CRU (Climate Research Unit) dataset(B).
(TIF)

Figure S8 **Similar as** **Figure 9A** **, but using temperature extracted from the CRU (Climate Research Unit) dataset.**
(TIF)

Figure S9 **Similar as** **Figure 3A** **, but using temperature extracted from the CRU (Climate Research Unit) dataset and the NDVI3 g from 1982 to 2010(A).** Similar as Figure 6, but using temperature extracted from the CRU (Climate Research Unit) dataset and the NDVI3 g from 1982 to 2010(B).
(TIF)

Figure S10 **Similar as** **Figure 9A** **, but using temperature extracted from the CRU (Climate Research Unit) dataset and the NDVI3 g from 1982 to 2010.**
(TIF)

Table S1 **Number of pixels in each land-cover class that experienced a significant** ***T*** **_eff_ increase from 1982 to 2008 (** ***P*** **<0.10).** The distribution of land-cover classes (C1–C23), defined according to the U.N. Land Cover Classification System, was determined from images obtained by the Medium Resolution Imaging Spectrometer [ESA GlobCover Project, led by MEDIAS-France, 48].
(DOCX)

Table S2 **The land cover classes used in Fig. S2A. Detailed definitions are given by** ***Hansen et al.*** [**80**] **.**
(DOCX)

Table S3 **Land-use types used in Fig. S2B, retrieved from the Global Landcover 2000 Web site** [**81**] **.**
(DOCX)
