# Supplementary figures and images for: Earlier-Season Vegetation Has Greater Temperature Sensitivity of Spring Phenology in Northern Hemisphere
Source: PLoS One. 2014 Feb 5;9(2):e88178. doi: 10.1371/journal.pone.0088178 (PMC3914920; doi:10.1371/journal.pone.0088178)

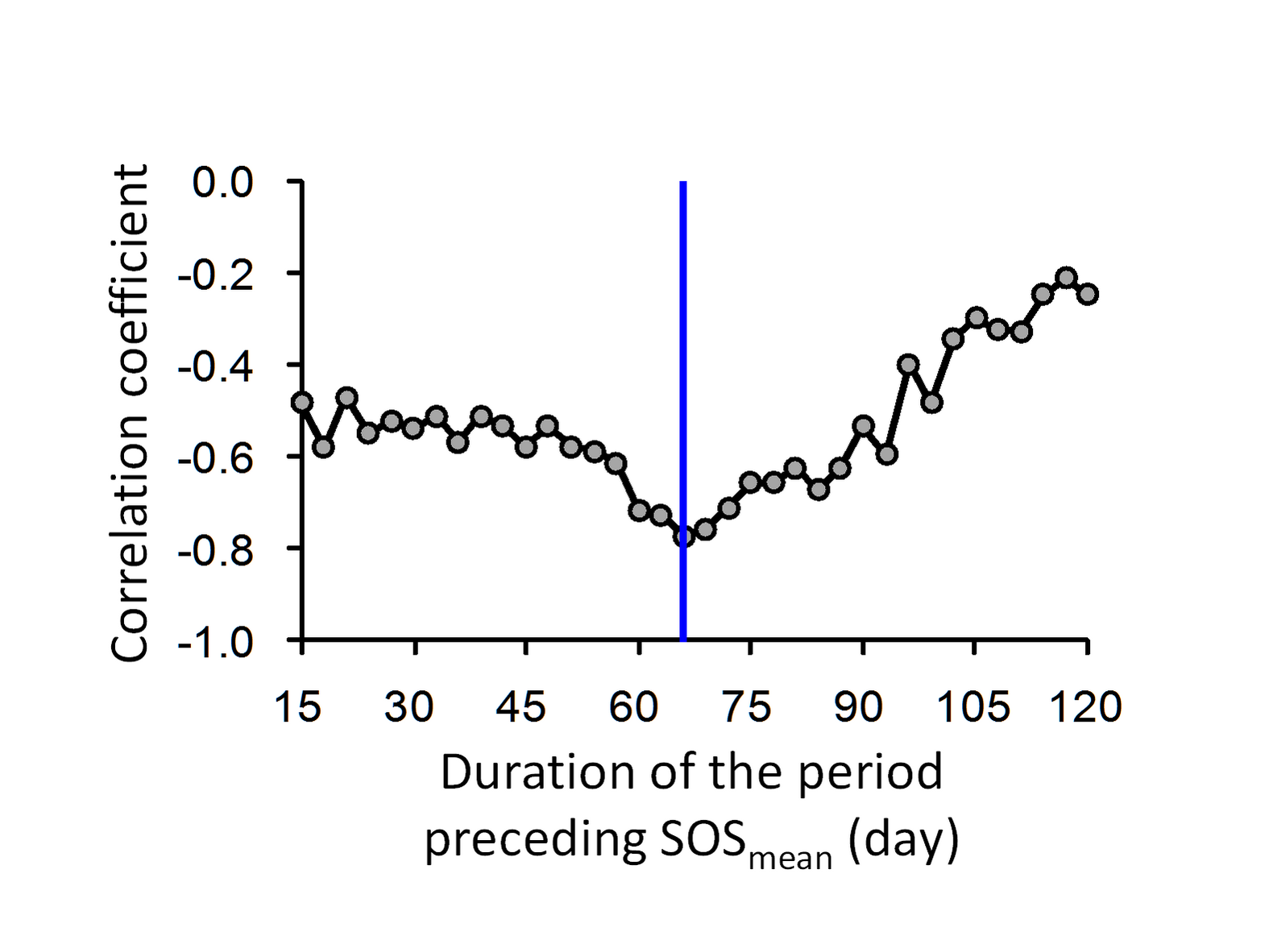

Supplement: Figure S1 — A schematic diagram indicating the determination of the duration of the preceding period with the potentially effective pre-season temperature. The x-axis gives the duration of the period preceding SOSmean of which the inter-annual variations in temperature are correlated (y-axis gives the correlation coefficient) to the inter-annual variations in SOS. In this case, the mean temperature of the 66-day period (the blue vertical line) preceding SOSmean is determined as the potentially effective pre-season temperature. (TIF) [file pone.0088178.s001.tif]

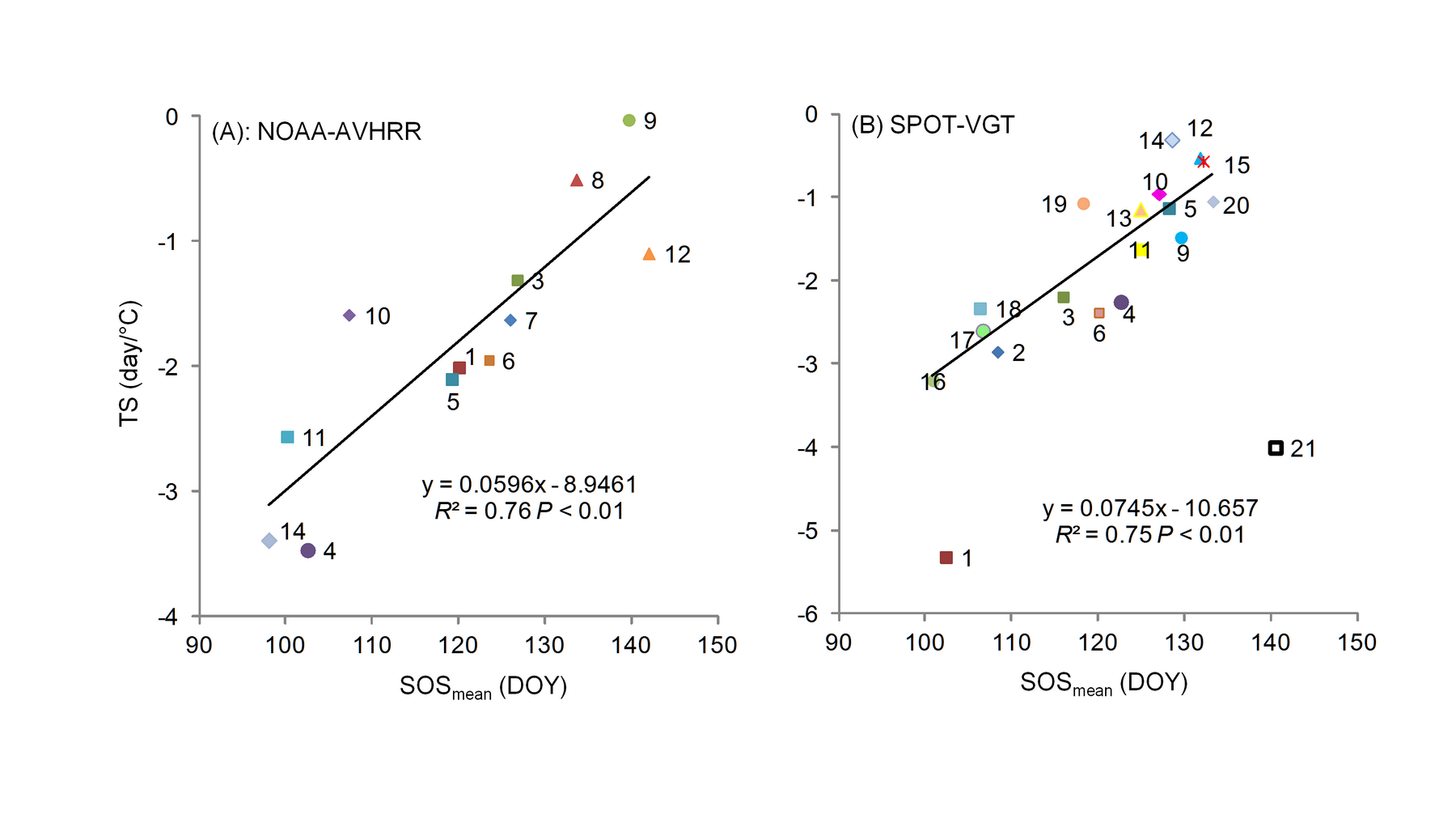

Supplement: Figure S2 — Temperature-sensitivity (TS) of the start of growing season (SOS) for different land-cover classes in relation to the mean SOS during 1982–2008 (SOSmean). Land covers are based on images obtained by NOAA-AVHRR and by SPOT-VGT (see Tables S2 and S3 for details). Land-cover types 1 and 21 in (B) were not included in the regression calculation. The values are the average of those in pixels with a significantly (P<0.10) negative SOS–Teff correlation and a significant (P<0.10) Teff increase. (TIF) [file pone.0088178.s002.tif]

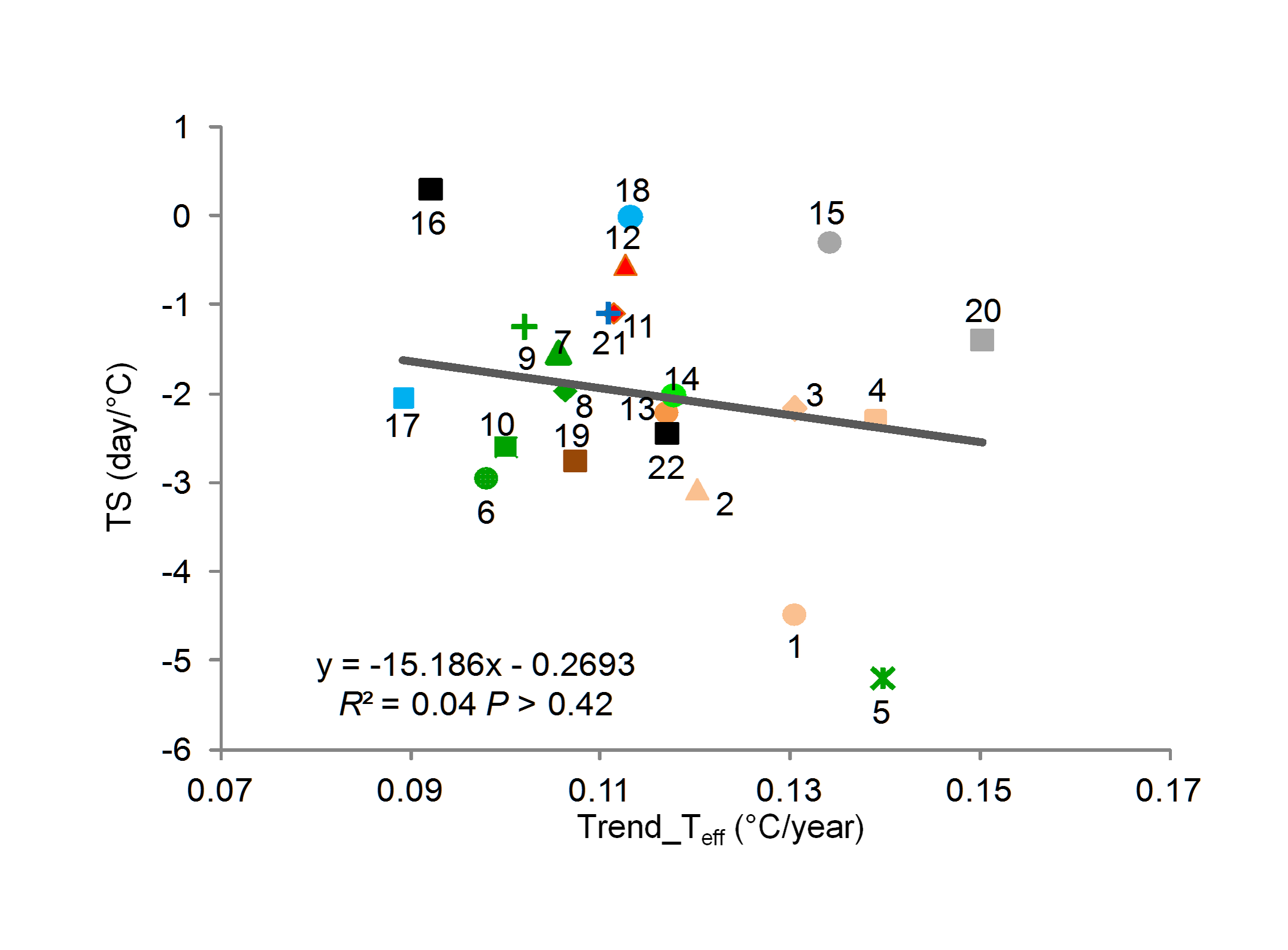

Supplement: Figure S3 — Relationship between the temperature-sensitivity (TS) of the SOS and the rate of change of the pre-season temperature ( T eff). See Figure 8 and Table S1 for the land-cover types. The values are the average of those in pixels with a significantly (P<0.10) negative SOS–T eff correlation and a significant (P<0.10) T eff increase. (TIF) [file pone.0088178.s003.tif]

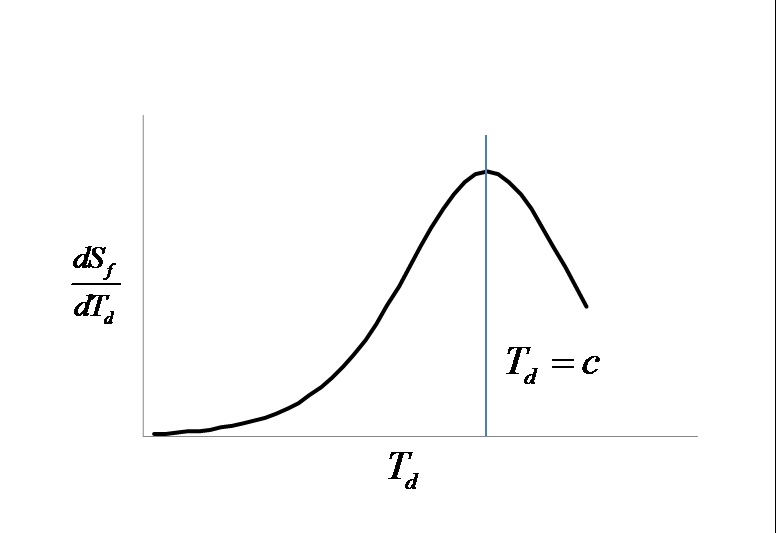

Supplement: Figure S4 — Relationship between and T d. In this example, b = –0.2 and c = 30. (TIF) [file pone.0088178.s004.tif]

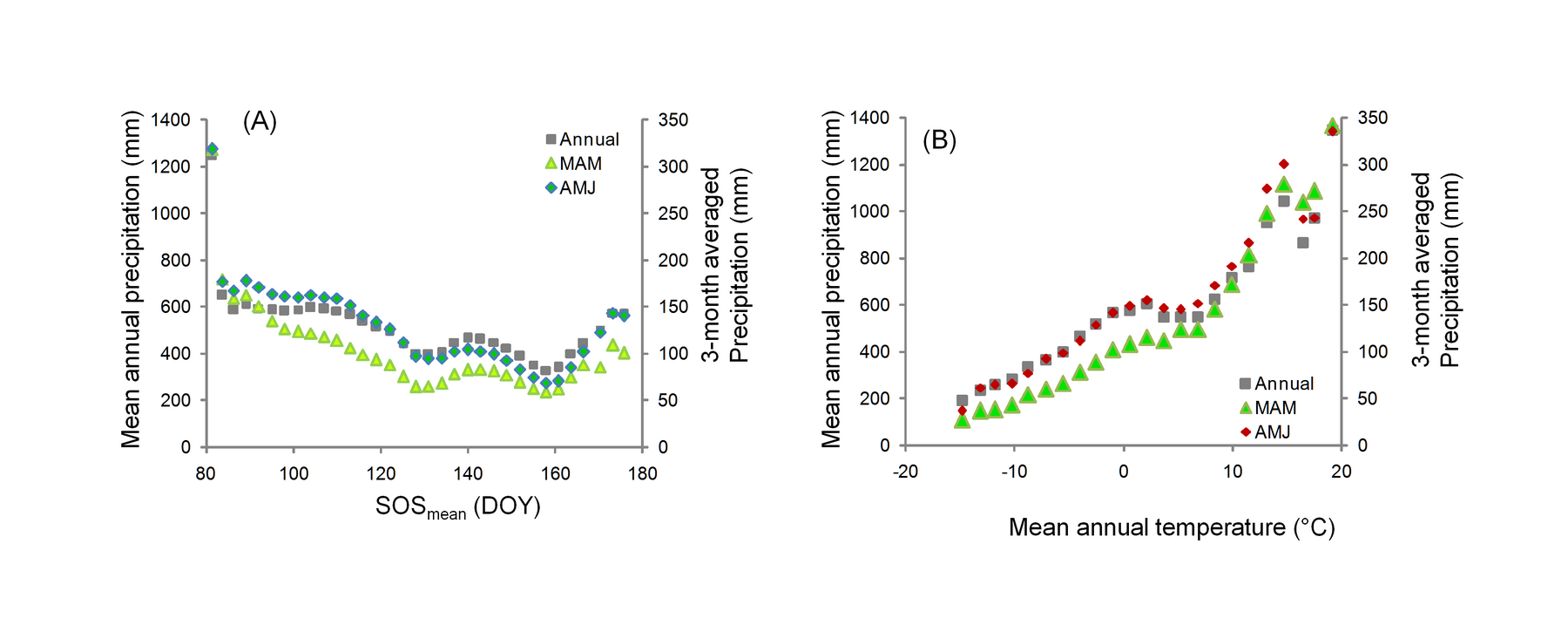

Supplement: Figure S5 — Mean annual, March–May (MAM), and April–June (AMJ) precipitation during 1982–2008 in relation to (A) SOSmean and (B) mean annual temperature. Only pixels with a positive change in T eff and a significantly (P<0.10) negative SOS–T eff correlation are included. Monthly temperature and precipitation data are from the CRU TS 3.2 data set (Mitchell TD and Jones PD, 2005, An improved method of constructing a database of monthly climate observations and associated high-resolution grids. Int J Climatol 25∶693–712.). (TIF) [file pone.0088178.s005.tif]

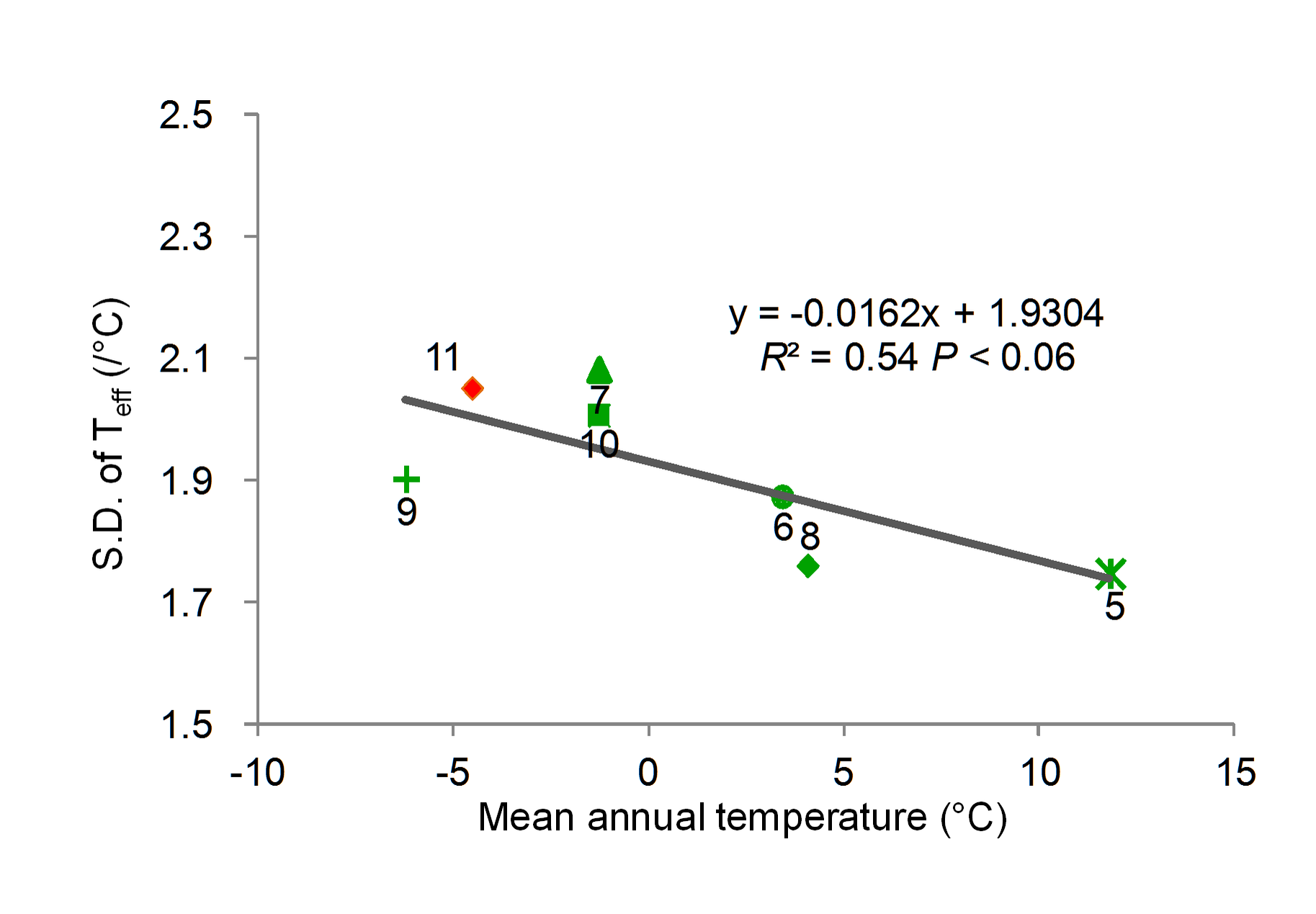

Supplement: Figure S6 — Relationship between the standard deviation (S.D.) of T eff and mean annual temperature for the forest land-cover classes. See Figure 9 for the land-cover types. The values are the average of those in pixels with a significantly (P<0.10) negative SOS–T eff correlation and a significant (P<0.10) T eff increase. (TIF) [file pone.0088178.s006.tif]

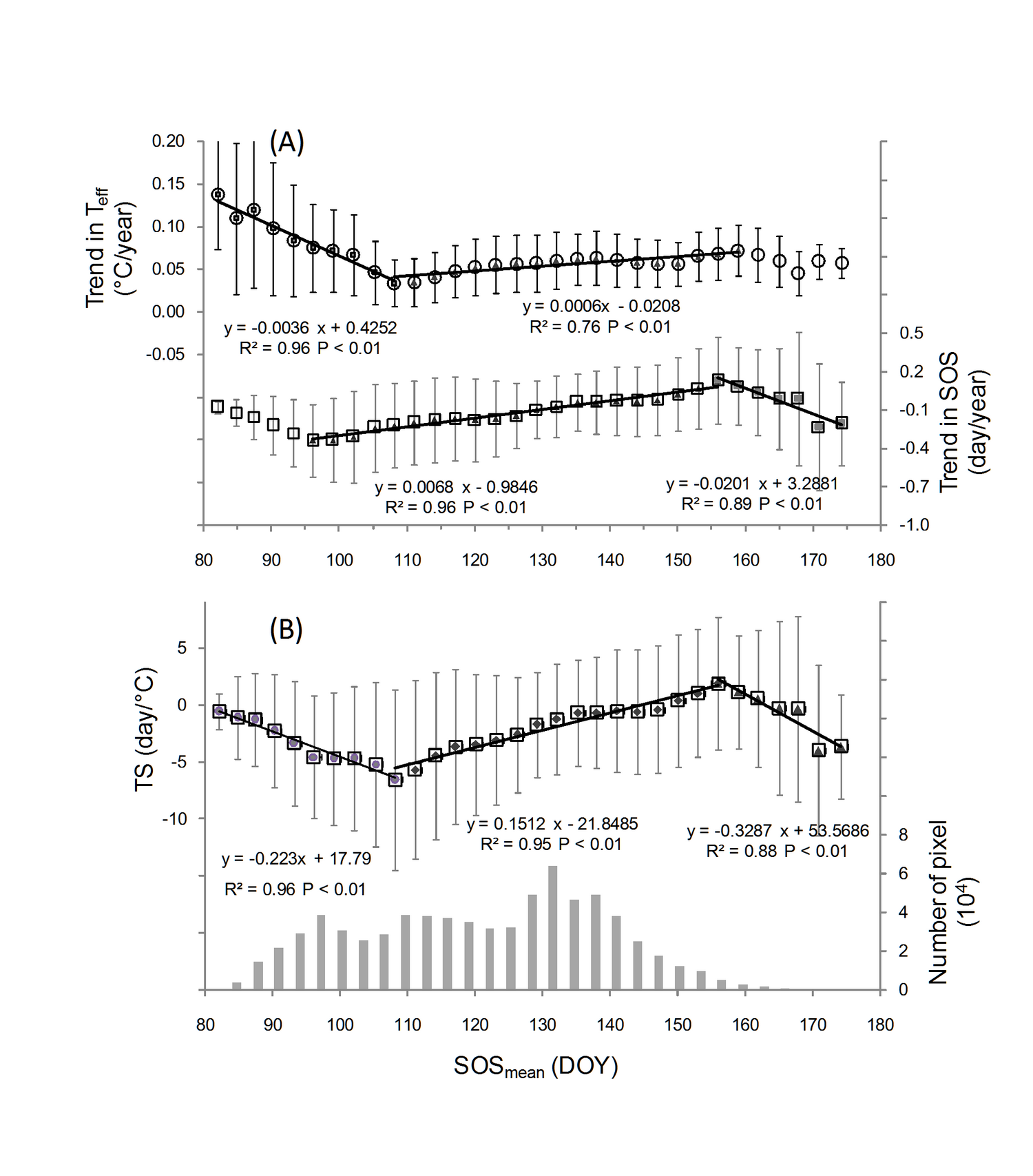

Supplement: Figure S7 — Similar as Figure 3A , but using temperature extracted from the CRU (Climate Research Unit) dataset (A). Similar as Figure 6, but using temperature extracted from the CRU (Climate Research Unit) dataset(B). (TIF) [file pone.0088178.s007.tif]

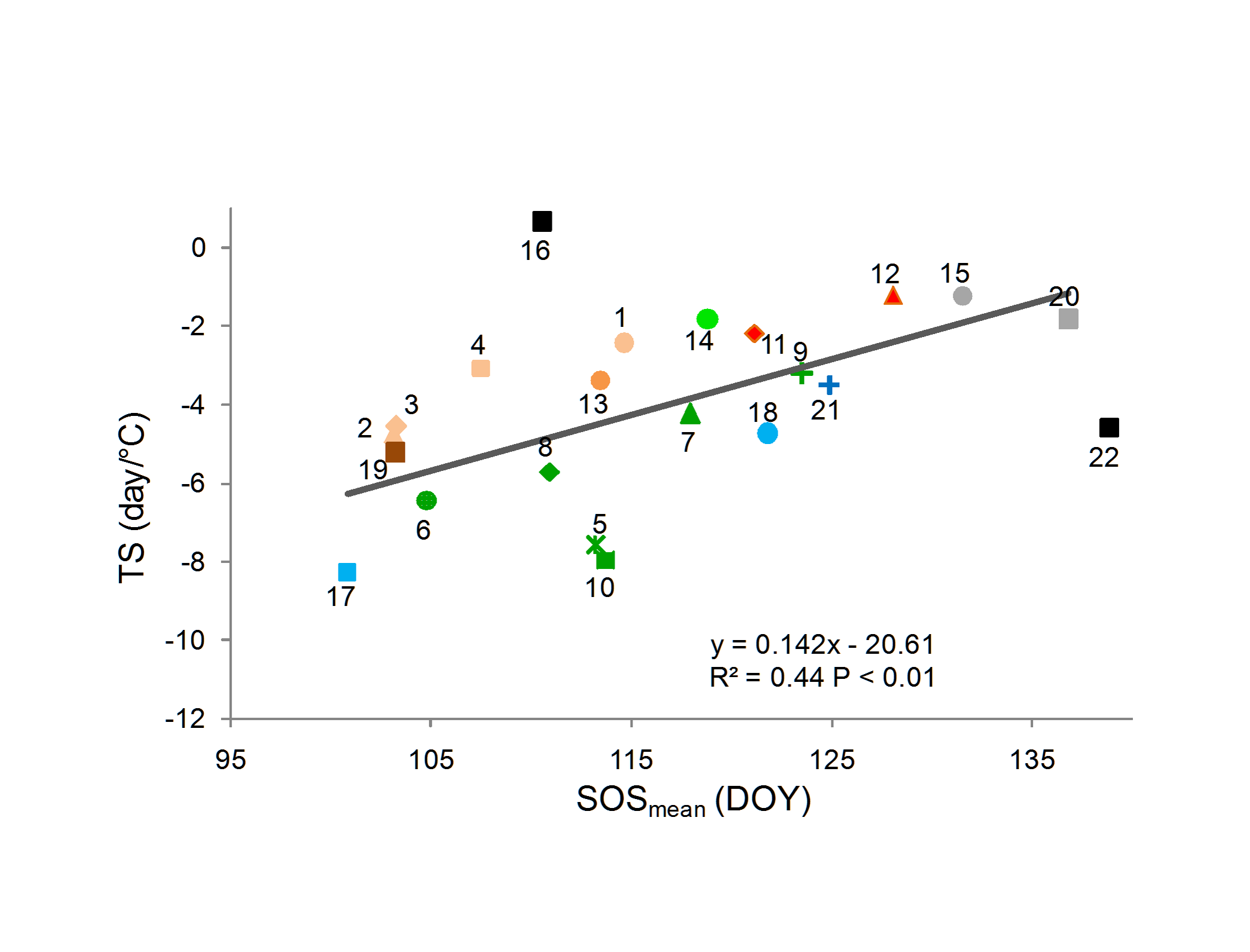

Supplement: Figure S8 — Similar as Figure 9A , but using temperature extracted from the CRU (Climate Research Unit) dataset. (TIF) [file pone.0088178.s008.tif]

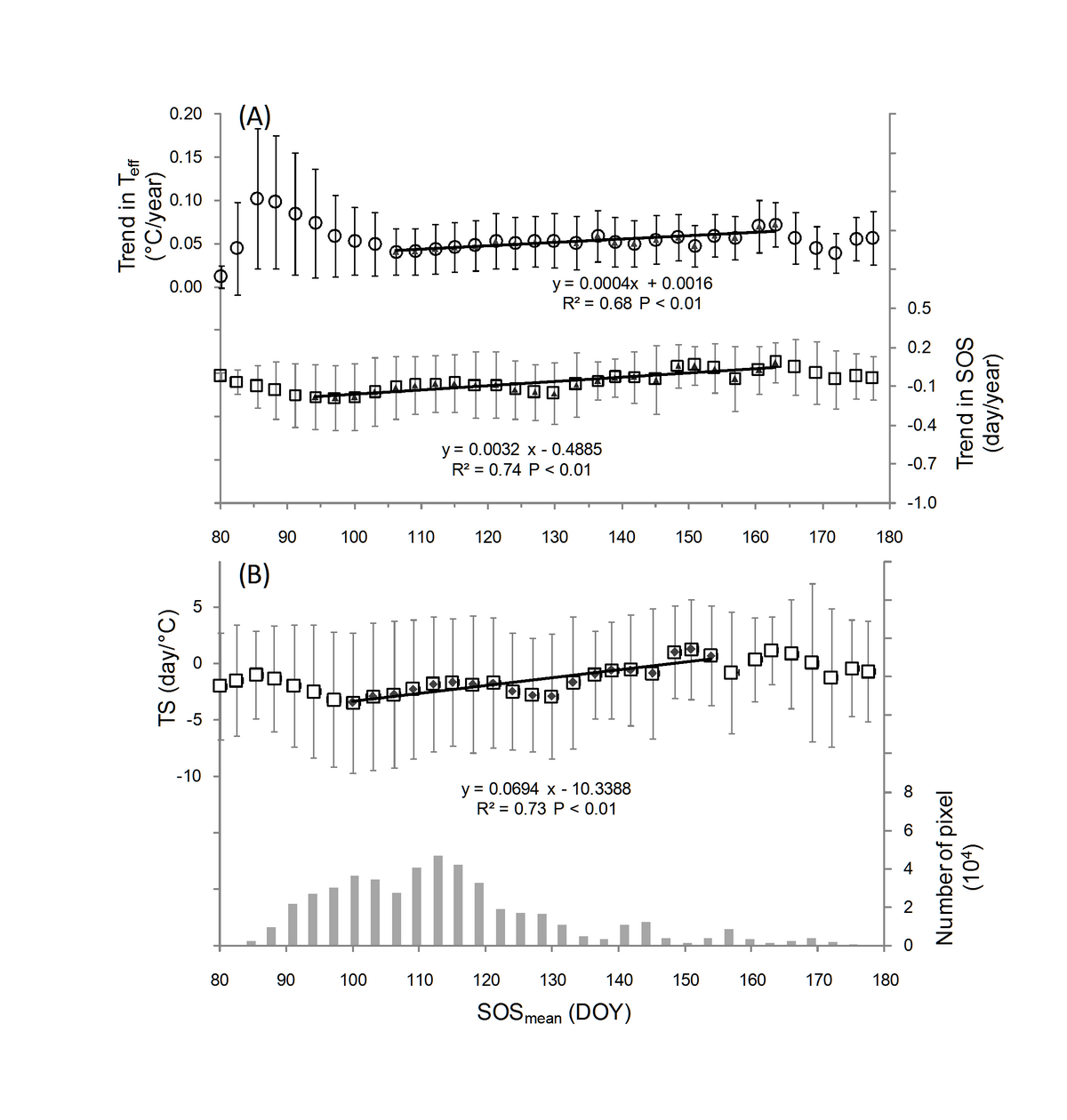

Supplement: Figure S9 — Similar as Figure 3A , but using temperature extracted from the CRU (Climate Research Unit) dataset and the NDVI3 g from 1982 to 2010(A). Similar as Figure 6, but using temperature extracted from the CRU (Climate Research Unit) dataset and the NDVI3 g from 1982 to 2010(B). (TIF) [file pone.0088178.s009.tif]

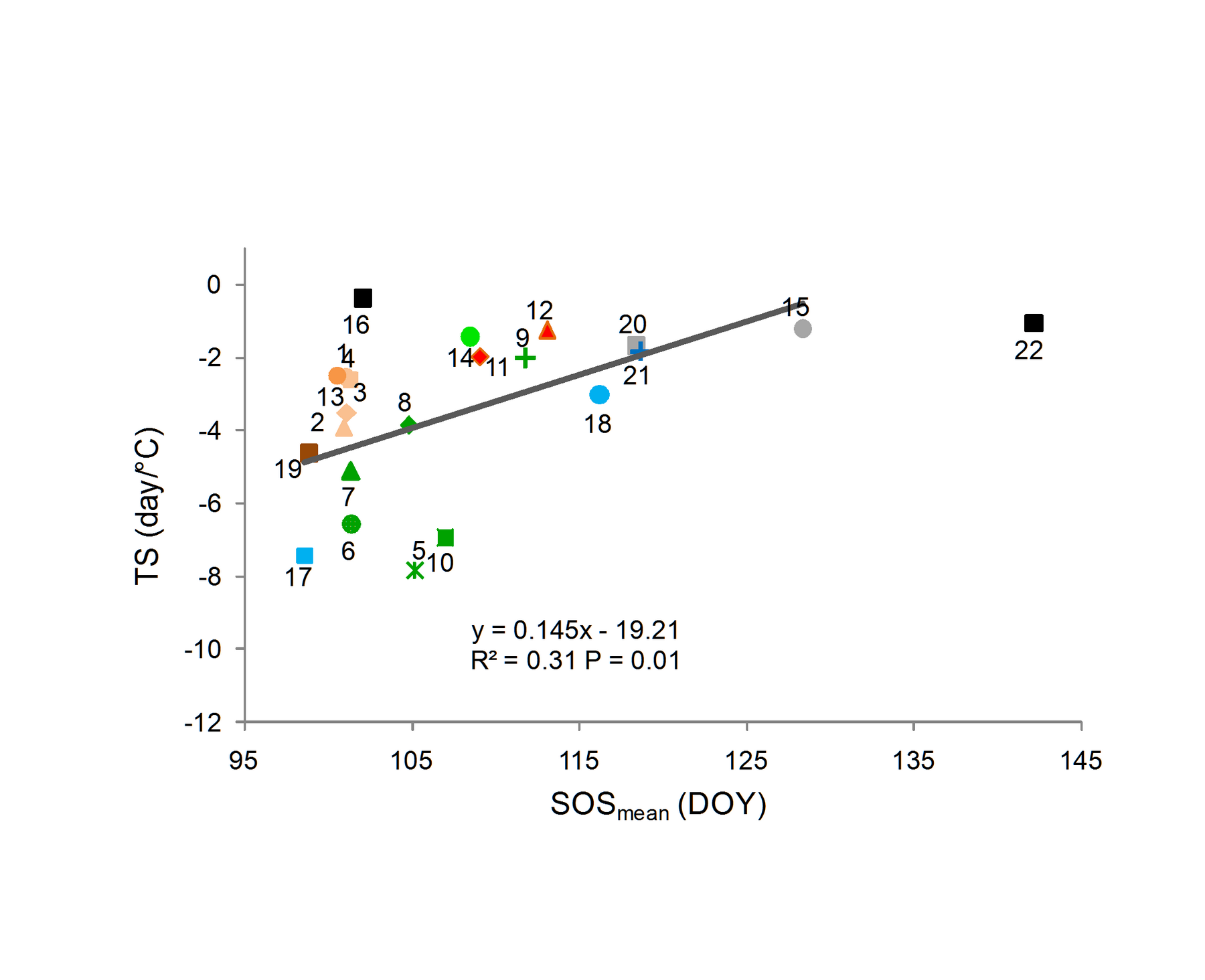

Supplement: Figure S10 — Similar as Figure 9A , but using temperature extracted from the CRU (Climate Research Unit) dataset and the NDVI3 g from 1982 to 2010. (TIF) [file pone.0088178.s010.tif]
