# Supplementary material for: Earlier-Season Vegetation Has Greater Temperature Sensitivity of Spring Phenology in Northern Hemisphere
Source: PLoS One. 2014 Feb 5;9(2):e88178. doi: 10.1371/journal.pone.0088178 (PMC3914920; doi:10.1371/journal.pone.0088178)
Supplement: Table S1 — Number of pixels in each land-cover class that experienced a significant T eff increase from 1982 to 2008 ( P <0.10). The distribution of land-cover classes (C1–C23), defined according to the U.N. Land Cover Classification System, was determined from images obtained by the Medium Resolution Imaging Spectrometer [ESA GlobCover Project, led by MEDIAS-France, 48]. (DOCX) [file pone.0088178.s011.docx]

**Table S1**.

|  | Abbreviated type names | Number of pixels | Land cover types definitions (*8*) |
| --- | --- | --- | --- |
| C1 | Irrigated croplands | 3660 | Post-flooding or irrigated croplands (or aquatic) |
| C2 | Rainfed croplands | 6343 | Rainfed croplands |
| C3 | Cropland dominated mosaics | 4107 | Mosaic cropland (50-70%) / vegetation (grassland/shrubland/forest) (20-50%) |
| C4 | Natural vegetation dominated mosaics | 10053 | Mosaic vegetation (grassland/shrubland/forest) (50-70%) / cropland (20-50%) |
| C5 | Closed to open broadleaved evergreen or semi-deciduous forest | 329 | Closed to open (>15%) broadleaved evergreen or semi-deciduous forest (>5m) |
| C6 | Closed broadleaved deciduous forest | 3043 | Closed (>40%) broadleaved deciduous forest (>5m) |
| C7 | Open broadleaved deciduous forest | 590 | Open (15-40%) broadleaved deciduous forest/woodland (>5m) |
| C8 | Closed needleleaved evergreen forest | 3279 | Closed (>40%) needleleaved evergreen forest (>5m) |
| C9 | Open needleleaved deciduous or evergreen forest | 67718 | Open (15-40%) needleleaved deciduous or evergreen forest (>5m) |
| C10 | Closed to open mixed forest | 4979 | Closed to open (>15%) mixed broadleaved and needleleaved forest (>5m) |
| C11 | Mosaic forest or shrubland / grassland | 16623 | Mosaic forest or shrubland (50-70%) / grassland (20-50%) |
| C12 | Mosaic grassland / forest or shrubland | 16372 | Mosaic grassland (50-70%) / forest or shrubland (20-50%) |
| C13 | Shrublands | 2639 | Closed to open (>15%) (broadleaved or needleleaved, evergreen or deciduous) shrubland (<5m) |
| C14 | Grasslands | 10119 | Closed to open (>15%) herbaceous vegetation (grassland, savannas or lichens/mosses) |
| C15 | Sparse vegetation | 52662 | Sparse (<15%) vegetation |
| C17 | Forest/woody wetland | 1 | Closed (>40%) broadleaved forest or shrubland permanently flooded - Saline or brackish water |
| C18 | Grassland/woody wetland | 2128 | Closed to open (>15%) grassland or woody vegetation on regularly flooded or waterlogged soil - Fresh, brackish or saline water |
| C19 | Urban areas | 222 | Artificial surfaces and associated areas (Urban areas >50%) |
| C20 | Bare areas | 11036 | Bare areas |
| C21 | Water bodies | 9393 | Water bodies |
| C22 | Permanent snow and ice | 2080 | Permanent snow and ice |
| C16 | Flooded broadleaved forest | 11 | Closed to open (>15%) broadleaved forest regularly flooded (semi-permanently or temporarily) - Fresh or brackish water |
| C23 | No data | 510 | No classification data |
