# Supplementary material for: Earlier-Season Vegetation Has Greater Temperature Sensitivity of Spring Phenology in Northern Hemisphere
Source: PLoS One. 2014 Feb 5;9(2):e88178. doi: 10.1371/journal.pone.0088178 (PMC3914920; doi:10.1371/journal.pone.0088178)
Supplement: Table S2 — The land cover classes used in Fig. S2A. Detailed definitions are given by Hansen et al. [80] . (DOCX) [file pone.0088178.s012.docx]

**Table S2.**

| 1 | Evergreen Needleleaf Forest |
| --- | --- |
| 2 | Evergreen Broadleaf Forest |
| 3 | Deciduous Needleleaf Forest |
| 4 | Deciduous Broadleaf Forest |
| 5 | Mixed Forest |
| 6 | Woodland |
| 7 | Wooded Grassland |
| 8 | Closed Shrubland |
| 9 | Open Shrubland |
| 10 | Grassland |
| 11 | Cropland |
| 12 | Bare Ground |
| 14 | Urban and Built |
