# Supplementary material for: Earlier-Season Vegetation Has Greater Temperature Sensitivity of Spring Phenology in Northern Hemisphere
Source: PLoS One. 2014 Feb 5;9(2):e88178. doi: 10.1371/journal.pone.0088178 (PMC3914920; doi:10.1371/journal.pone.0088178)
Supplement: Table S3 — Land-use types used in Fig. S2B, retrieved from the Global Landcover 2000 Web site [81] . (DOCX) [file pone.0088178.s013.docx]

**Table S3.**

| 1 | Tree cover, broadleaved, evergreen LCCS > 15% tree cover, tree height > 3 m (examples of subclasses at regional level: closed > 40% tree cover; open 15–40% tree cover) |
| --- | --- |
| 2 | Tree cover, broadleaved, deciduous, closed |
| 3 | Tree cover, broadleaved, deciduous, open (open 15–40% tree cover) |
| 4 | Tree cover, needle-leaved, evergreen |
| 5 | Tree cover, needle-leaved, deciduous |
| 6 | Tree cover, mixed leaf type |
| 7 | Tree cover, regularly flooded, fresh water (and brackish) |
| 8 | Tree cover, regularly flooded, saline water (daily variation of water level) |
| 9 | Mosaic: tree cover/other natural vegetation |
| 10 | Tree cover, burnt |
| 11 | Shrub cover, closed–open, evergreen (examples of subclasses at regional level: (i) sparse tree layer) |
| 12 | Shrub cover, closed–open, deciduous (examples of subclasses at regional level: (i) sparse tree layer) |
| 13 | Herbaceous cover, closed–open (examples of subclasses at regional level: (i) natural, (ii) pasture, (iii) sparse trees or shrubs) |
| 14 | Sparse herbaceous or sparse shrub cover |
| 15 | Regularly flooded shrub and/or herbaceous cover |
| 16 | Cultivated and managed areas (examples of subclasses at regional level: (i) terrestrial; (ii) aquatic (= flooded during cultivation), and under terrestrial: (iii) tree crop and shrubs (perennial), (iv) herbaceous crops (annual), non-irrigated, (v) herbaceous crops (annual), irrigated) |
| 17 | Mosaic: cropland/tree cover/other natural vegetation |
| 18 | Mosaic: cropland/shrub or grass cover |
| 19 | Bare areas |
| 20 | Water bodies (natural and artificial) |
| 21 | Snow and ice (natural and artificial) |
| 22 | Artificial surfaces and associated areas |
